# Prognostic Nomogram for Predicting Lower Extremity Deep Venous Thrombosis in Neurointensive Care Unit Patients: A Prospective Observational Study

**DOI:** 10.3389/fneur.2021.761029

**Published:** 2022-01-28

**Authors:** Rongqing Li, Jinxia Jiang, Yu Song, Jianan Zhang, Yawen Wu, Lingzhi Wu, Xiaoping Zhu, Li Zeng

**Affiliations:** ^1^Neurointensive Care Unit, Shanghai Tenth People's Hospital, School of Medicine, Tongji University, Shanghai, China; ^2^Department of Emergency, Shanghai Tenth People's Hospital, School of Medicine, Tongji University, Shanghai, China; ^3^Department of Neurosurgery, Shanghai Tenth People's Hospital, School of Medicine, Tongji University, Shanghai, China; ^4^Department of Nursing, Shanghai Tenth People's Hospital, School of Medicine, Tongji University, Shanghai, China

**Keywords:** neurointensive care unit, lower extremity, deep venous thrombosis, prediction model, nomogram

## Abstract

**Background:**

Deep venous thrombosis (DVT) of the lower extremities is one of the common complications for neurointensive care unit patients, which leads to increased morbidity and mortality. The purpose of our study was to explore risk factors and develop a prognostic nomogram for lower extremity DVT in neurointensive care unit patients.

**Methods:**

We prospectively collected and analyzed the clinical data of 420 neurointensive care unit patients who received treatment in our institution between January 2018 and September 2019. Stepwise logistic regression was used to select predictors. R software was used to develop the prognostic nomogram. The performance of the nomogram was validated using a validation cohort of patients with data collected between October 2019 and March 2020.

**Results:**

Among 420 patients, 153 (36.4%) had lower extremity DVT and five (1.2%) had both DVT and pulmonary embolism (PE) in our study. Logistic regression analysis indicated that age [odds ratio (OR): 1.050; 95% confidence interval (CI): 1.029–1.071; *P* < 0.001], Glasgow Coma Scale (GCS) score (OR: 0.889; 95% CI: 0.825–0.959; *P* = 0.002), D-dimer level (OR: 1.040; 95% CI: 1.008–1.074; *P* = 0.014), muscle strength (OR: 2.424; 95% CI: 1.346–4.366; *P* = 0.003), and infection (OR: 1.778; 95% CI: 1.034–3.055; *P* = 0.037) were independent predictors for lower extremity DVT. These predictors were selected to be included in the nomogram model. The area under the curve values in the primary cohort and validation cohort were 0.817 (95% CI: 0.776–0.858) and 0.778 (95% CI: 0.688–0.868), respectively, and respective Brier scores were 0.167 and 0.183.

**Conclusion:**

Age, GCS score, D-dimer level, muscle strength, and infection are independent predictors for lower extremity DVT. The nomogram is a reliable and convenient model to predict the development of lower extremity DVT in neurointensive care unit patients.

## Introduction

Patients admitted to the neurointensive care unit are at a high risk for lower extremity deep vein thrombosis (DVT) due to factors such as disturbance of consciousness, craniotomy, paralysis, and maintaining a long-term bedridden state (1–4). Studies have shown that the incidence can be as high as 43–50% (5, 6). Lower extremity DVT not only causes swelling, localized pain, and varicose veins of the affected limb, but it may also lead to pulmonary embolism (PE), for which it is the primary cause. According to literature reports in the past 10 years, PE is a leading cause of death in neurointensive care unit patients, with a mortality rate between 9 and 50% (4). Therefore, early assessment and prophylaxis should be administered to reduce its occurrence and subsequent mortality. The nomogram model can integrate relevant risk factors and individually predict the risk of adverse clinical events. It has been widely used in medical treatments, especially in clinical predictive models (7). Despite the risks of DVT and subsequent PE, there is still no nomogram model to predict the occurrence of lower extremity DVT in neurointensive care units. In this prospective study, we focused on neurointensive care unit patients and established a reliable prognostic nomogram for providing a reference to identify the patients at high-risk of developing lower extremity DVT early.

## Methods

### Patients

**Figure 1:**
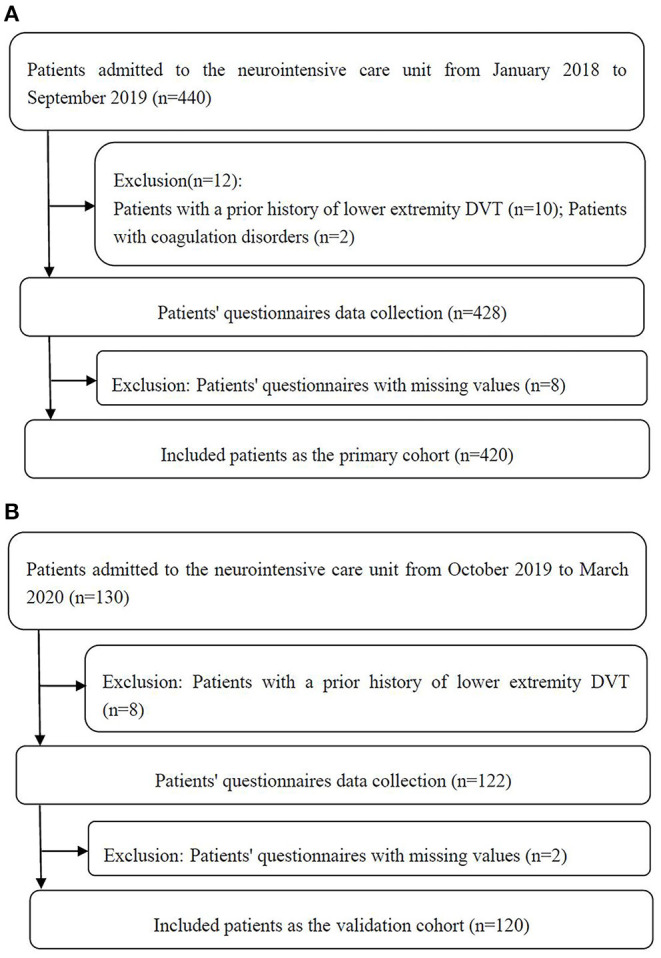
Flowchart of the primary cohort **(A)** and validation cohort **(B)**.

We conducted a prospective observational study to collect data from neurointensive care unit patients who received treatment in our institution between January 2018 and September 2019. These patients were included in the primary cohort. Additional data were collected from patients who were admitted to the neurointensive care unit between October 2019 and March 2020 to form a validation cohort. The study protocol was approved by the Ethics Committees of our institution, and we obtained informed consent from all participants. Inclusion criteria were as follows: (1) age ≥18 years and (2) admitted to the neurointensive care unit for ≥24 h. The exclusion criteria were as follows: (1) patients with a prior history of lower extremity DVT and/or PE and (2) patients with coagulation disorders. Figure 1 shows the flowchart of the screening process in detail.

When patients are admitted to the neurointensive care unit, the surgeons will first assess the patients' risk of bleeding, before administering individual prophylaxis for lower extremity DVT; this may include mechanical prophylaxis, such as compression stockings, intermittent pneumatic compression, and ankle pump activities, among others; pharmacological prophylaxis, such as individualized application of low molecular weight heparin (i.e., enoxaparin sodium, 2,000 or 4,000 IU every 8–12 h); or a combination of these methods. Patients who have a high risk of rebleeding (such as patients with craniectomy, traumatic brain injury patients, or patients with intracranial hemorrhage, etc.) are first administered mechanical prophylaxis before being administered pharmacological prophylaxis after the acute phase of bleeding. Once patients develop lower extremity DVT, we immediately stop the use of intermittent pneumatic compression, raise and immobilize the affected limb, adjust the dose of anticoagulants, and use preventive inferior vena cava filters when necessary.

### Diagnosis of Lower Extremity DVT

Lower extremity DVT can be diagnosed by auxiliary tests such as Doppler ultrasonography, magnetic resonance imaging venography, and angiography (8); among these methods, ultrasonography is the most accurate non-invasive examination for the diagnosis of lower extremity DVT (9). In our institution, a complete duplex ultrasound examination is performed to diagnose lower extremity DVT. Considering the high incidence of lower extremity DVT in the neurointensive care unit, examinations are routinely performed every Monday, Wednesday, and Friday.

### Data Collection and Acquisition

A self-designed questionnaire was used to collect the data, which were compiled by literature research and expert group meetings. The questionnaire consisted of three parts: (1) patients' demographics, such as sex, age, etc.; (2) medical history, such as hypertension, diabetes, etc.; and (3) clinical features, including diagnosis, Glasgow Coma Scale (GCS) score, Caprini score, Acute Physiology and Chronic Health Evaluation II (APACHE II) score, muscle strength when entering the neurointensive care unit, days of stay in the neurointensive care unit, whether the patient had surgery, D-dimer levels after the third day of surgery or conservative treatment, whether mechanical ventilation was used for more than 48 h, central venous catheter (CVC), infection (intracranial infection, catheter-related bloodstream infection, urinary tract infection, or pneumonia), and use of vasopressors, etc. Muscle strength was transformed into a categorical variable (≥ grade 4 and ≤ grade 3). The data of this study were collected by five intensive care unit-qualified graduate students through a computerized clinical information system, and then the data were extracted. Incomplete information questionnaires were discarded.

### Statistical Analysis

Statistical analyses were performed using SPSS 23.0 (IBM Corp., Armonk, NY, USA). The categorical variables and continuous variables are separately expressed as numbers (frequencies) and medians (interquartile ranges, IQRs). The Shapiro-Wilk test was used to check normal distribution for continuous variables. The Mann-Whitney *U*-test and chi-square test were used to assess continuous and categorical variables, respectively. The following statistical analysis only included variables with a *P* < 0.05. Logistic regression analysis was then conducted to determine the independent predictors for lower extremity DVT, and backward and forward stepwise regressions were performed to select the best model. Subsequently, according to the results of logistic regression, the nomogram was formulated in R software 4.0.5 (R Foundation for Statistical Computing, Vienna, Austria). The receiver operating characteristic curve (ROC) was plotted and area under the ROC curve (AUC) was determined to evaluate the discrimination of the nomogram. The Brier score and calibration curve with bootstraps of 1,000 resamples were used to evaluate the calibration of the nomogram model. The decision curve analysis was plotted to measure the clinical benefit of the nomogram. A two-sided test was used, and *P* < 0.05 was considered statistically significant.

## Results

### Demographic and Clinical Characteristics

A total of 420 patients were included in the primary cohort and 120 patients in the validation cohort. Patient age ranged from 18 to 91 years (53.96 ± 15.51). Among the primary cohort patients, 153 patients had lower extremity DVT, leading to an incidence rate of 36.4%. Moreover, 54.9% of patients had calf muscle vein thrombosis; of these, five patients were complicated by PE, and two patients died of PE.

Demographics, clinical, and laboratory characteristics for both cohorts are shown in Table 1. As the APACHE-II score includes the age, surgery condition, and GCS score of the patients, the four APACHE-II score subsets are listed separately to avoid duplication of variables. Patients in the validation cohort had lower GCS scores than patients in the primary cohort [median (IQR), 6 (4–9) vs. 7 (5–11); *P* = 0.007]. No other significant differences were noted between the cohorts.

**Table 1:** Baseline clinical and demographic characteristics of the primary and validation cohorts.

**Parameters**	**Primary cohort**	**Validation cohort**	***P*** **-value**
Age (years), median (IQR)	56.0 [43.2, 65.0]	54.0 [41.0, 64.0]	0.279
Sex (men), *n* (%)	257 (61.2)	78 (65.0)	0.142
Diagnostic category, *n* (%)			0.333
Neurovascular disease	148 (35.2)	43 (35.9)	
Central nervous system tumor	146 (34.8)	45 (37.5)	
Traumatic brain injury	96 (22.9)	28 (23.3)	
Others	30 (7.1)	4 (3.3)	
GCS score, median (IQR)	7.0 [5.0, 11.0]	6.0 [4.0, 9.0]	0.007
APACHE-II score, median (IQR)	16.0 [11.0, 20.0]	17.5 [13.0, 21.0]	0.124
APACHE-II A	3.0 [0.0, 5.0]	3.0 [2.0,5.0]	0.272
APACHE-II B	5.0 [2.0, 5.0]	5.0 [2.0, 5.0]	0.152
APACHE-II C	4.0 [8.0, 10.0]	6.0 [9.0, 11.0]	0.062
APACHE-II D	2.0 [1.0, 4.0]	3.0 [1.2, 4.0]	0.957
Caprini score, median (IQR)	9.0 [7.0, 12.0]	10.0 [7.0, 12.7]	0.109
NICU stay, median (IQR)	14.0 [5.0, 24.0]	14.0 [6.2, 25]	0.996
D-dimer level (μg/mL), median (IQR)	2.8 [1.5, 8.5]	2.8 [1.3, 8.1]	0.256
Muscle strength, *n* (%)			0.352
≤ 3 grade	207 (49.3)	53 (44.2)	
Hypertension, *n* (%)	183 (43.6)	51 (42.5)	0.917
Diabetes, *n* (%)	59 (14)	20 (16.7)	0.467
Surgery, *n* (%)	335 (79.8)	91 (75.8)	0.375
CVC, *n* (%)	398 (94.8)	113 (94.2)	0.819
Hemostatic drugs, *n* (%)	361 (86)	103 (85.8)	1.000
Vasopressors, *n* (%)	65 (15.5)	14 (11.7)	0.379
Sedative drugs, *n* (%)	366 (87.1)	101 (84.2)	0.449
Mechanical ventilation (≥48 h),	162 (38.6)	41 (34.2)	0.395
Infection, *n* (%)	188 (44.8)	61 (53.5)	0.112

*APACHE, Acute Physiology and Chronic Health Evaluation. Continuous variables are presented as medians and interquartile ranges; categorical variables are presented as frequencies*.

### Risk Factors for Lower Extremity DVT

Results of the univariate analysis of patients in the primary cohort with and without lower extremity DVT are shown in Table 2. Among all the variables, the following variables had significant differences between the two groups (*P* < 0.05): age, diagnostic category, GCS score, APACHE II score, Caprini score, days of stay, D-dimer level, muscle strength, hypertension, vasopressors, mechanical ventilation, and infection. Based on these variables, the dichotomous logistic regression analysis was then performed. The results showed that age, GCS score, D-dimer level, muscle strength, and infection were independent risk factors in patients who developed lower extremity DVT (Table 3).

**Table 2:** Results of univariate analysis of risk factors for lower extremity DVT in the primary cohort.

	**Lower extremity DVT positive (*N* = 153)**	**Non-lower extremity DVT negative (*N* = 267)**	***P*** **-value**
Age (years), median (IQR)	60.0 [54.0, 66.0]	52.0 [38.0, 64.0]	<0.001
Sex (men), *n* (%)	95 (62.1)	162 (60.7)	0.774
Diagnostic category, *n* (%)			0.012
Neurovascular disease	59 (38.6)	89 (33.4)	
Central nervous system tumor	41 (26.8)	105 (39.3)	
Traumatic brain injury	45 (29.4)	51 (19.1)	
Others	8 (5.2)	22 (8.2)	
GCS score, median (IQR)	6.0 [3.0, 8.0]	9.0 [6.0, 15.0]	<0.001
APACHE-II score, median (IQR)	19.0 [15.0, 23.0]	14.0 [8.0, 18.0]	<0.001
APACHE-II A	3.0 [2.0, 5.0]	2.0 [0.0, 3.0]	<0.001
APACHE-II B	5.0 [2.0, 5.0]	2.0 [2.0, 5.0]	0.008
APACHE-II C	9.0 [7.0, 12.0]	6.0 [0.0, 9.0]	<0.001
APACHE-II D	3.0 [1.5, 5.0]	2.0 [0.0, 4.0]	0.008
Caprini score, median (IQR)	11.0 [8.0, 13.0]	8.0 [7.0, 12.0]	<0.001
NICU stay, median (IQR)	18.0 [9.5, 29.0]	10.0 [5.0, 21.0]	<0.001
D-dimer level (μg/mL), median (IQR)	4.0 [2.1, 13.3]	2.5 [1.3, 6.1]	<0.001
Muscle strength, *n* (%)			<0.001
≥4 grade	40 (26.1)	137 (64.8)	
≤ 3 grade	113 (73.9)	94 (35.2)	
Hypertension, *n* (%)	80 (52.3)	103 (38.6)	0.006
Diabetes, *n* (%)	19 (12.4)	40 (15)	0.467
Surgery, *n* (%)	120 (78.4)	215 (80.5)	0.607
CVC, *n* (%)	147 (96.1)	251 (85)	0.359
Hemostatic drugs, *n* (%)	134 (87.6)	227 (85)	0.467
Vasopressors, *n* (%)	36 (23.5)	29 (10.9)	0.001
Sedative drugs, *n* (%)	137 (89.5)	229 (85.8)	0.266
Mechanical ventilation (≥48 h),	85 (55.6)	77 (10.9)	<0.001
Infection, *n* (%)	95 (62.1)	93 (34.8)	<0.001

*APACHE, Acute Physiology and Chronic Health Evaluation. Continuous variables are presented as medians and interquartile ranges; categorical variables are presented as frequencies*.

**Table 3:** Results of logistic regression analysis of predictors for lower extremity DVT.

**Variables**	**B**	**SE**	***P*** **-value**	**OR**	**95% CI**
Age	0.49	0.010	<0.001	1.050	1.029–1.071
GCS score	−0.117	0.039	0.002	0.889	0.825–0.959
D-dimer level	0.040	0.016	0.014	1.040	1.008–1.074
Muscle strength	0.885	0.300	0.003	2.424	1.346–4.366
Infection	0.575	0.276	0.037	1.778	1.034–3.055
Constant	−3.620	0.786	<0.001	0.027	—

*SE, standard error; OR, odds ratio; CI, confidence interval; GCS, Glasgow Coma Scale*.

### Development and Validation of the Nomogram for Lower Extremity DVT

**Figure 2:**
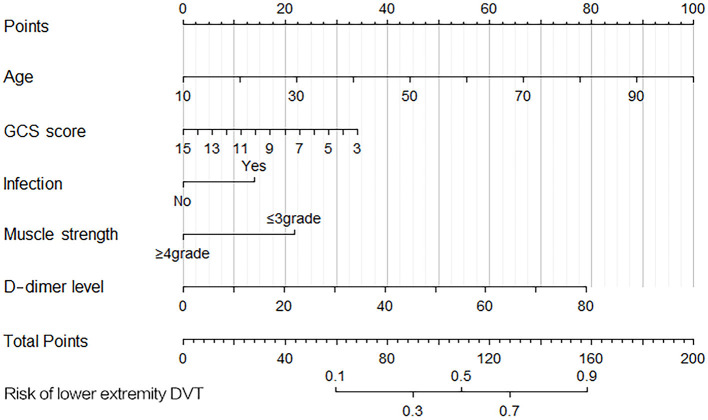
Nomogram for predicting the risk of lower-extremity deep vein thrombosis (DVT). Points were assigned for age, D-dimer level, Glasgow Coma Scale (GCS) score, muscle strength, and infection. The total score obtained by adding up the scores of all individual variables is used to find the appropriate position on the “Risk of DVT” axis to determine the patient's individual risk of lower extremity DVT. A simple example analysis: A 50-year-old patient (~45 points) has a GCS score of 8 (20 points) and muscle strength of 3 grade (22 points) when admitted to the neurointensive care unit. After laboratory tests, his blood D-dimer level was 10 μg/mL (10 points) after the third day of surgery, and he had no infection (0 points) when he was admitted to the neurointensive care unit. The total score for this patient was 97 points. According to the nomogram, the risk of lower extremity DVT in this patient is ~36%.

The prognostic nomogram that integrated all significant independent factors is shown in Figure 2. In this model, age exhibited the greatest influence on lower extremity DVT, followed by D-dimer level, GCS score, muscle strength, and infection, respectively. The total score of the five independent prognostic factors in the nomogram model was positively correlated with the patients' risk of developing lower extremity DVT.

**Figure 3:**
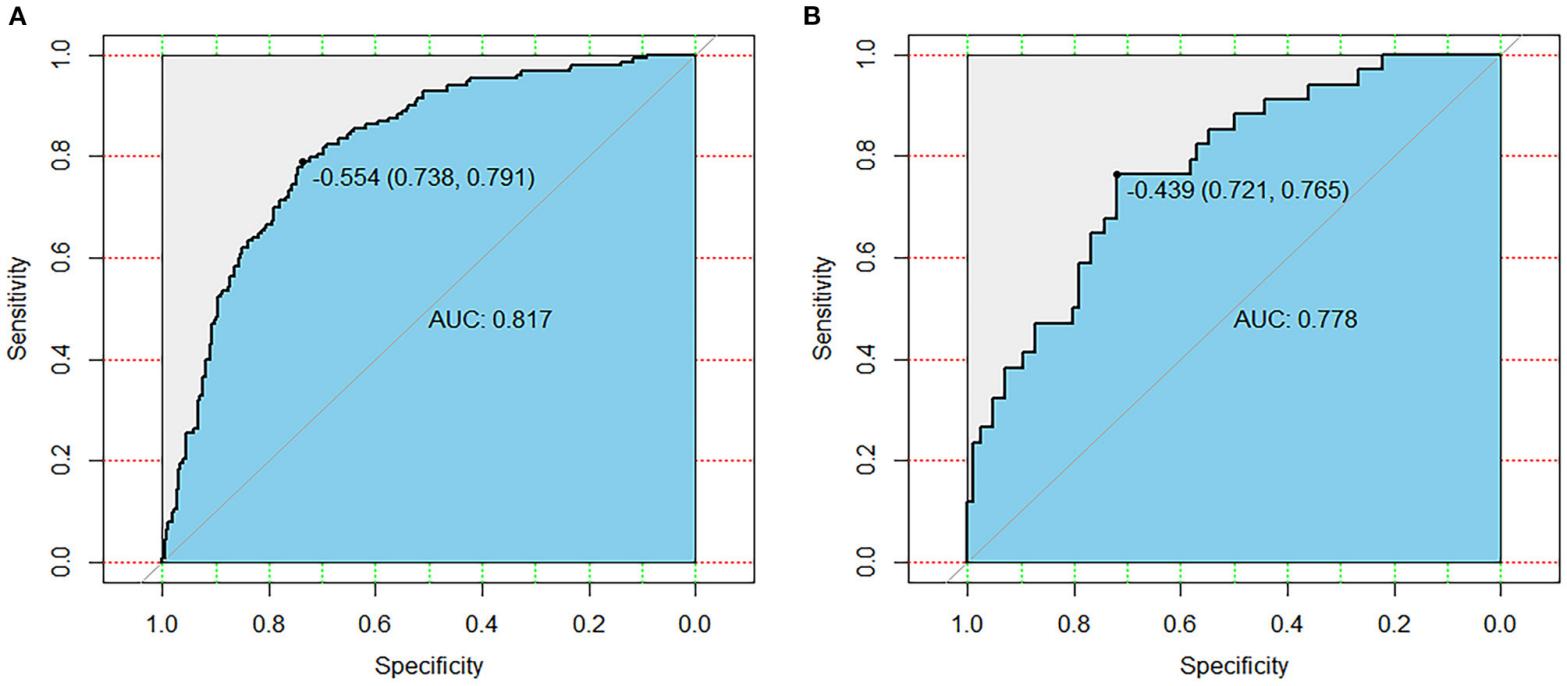
Receiver operating characteristic curves of the primary cohort **(A)** and validation cohort **(B)**. AUC, area under the receiver operating characteristic curve.

To evaluate the discrimination performance of the nomogram model, we calculated the AUC. The AUC values of this nomogram model were 0.817 [95% (CI): 0.776–0.858, Figure 3A] in the primary cohort and 0.778 (95% CI: 0.688–0.868, Figure 3B) in the validation cohort. We also performed 10-fold cross-validation by using all the data to verify the predictability of the nomogram, and the maximum AUC was 0.896 (95% CI: 0.789–1), indicating that this nomogram model was discriminatory.

**Figure 4:**
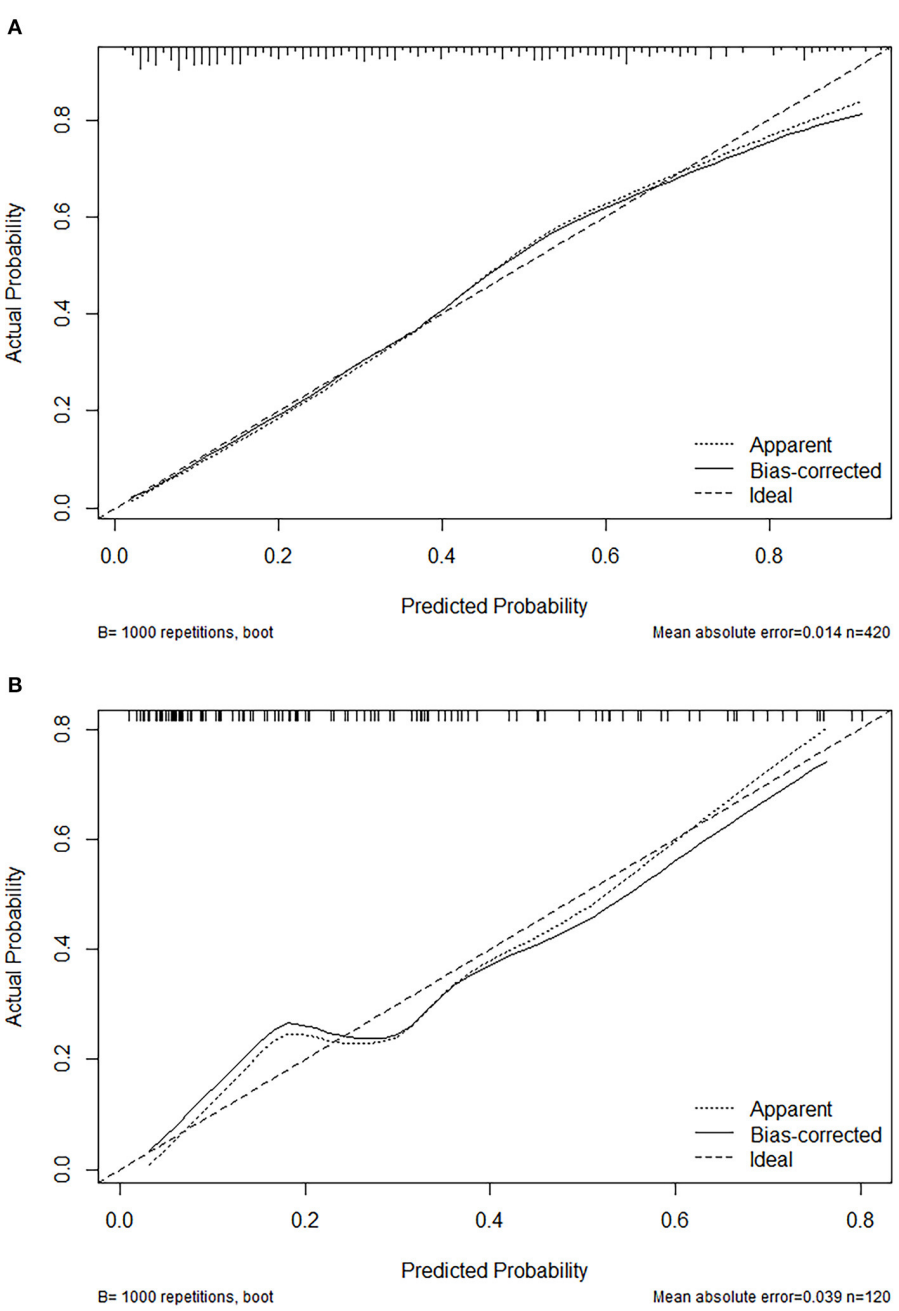
Calibration plot of the nomogram in the primary cohort **(A)** and validation **(B)** cohort. Predictions generated from the model are plotted against actual patient outcomes. The 45-degree line represents the perfect model calibration. The dotted line (apparent) indicates calibration when the model is applied to each set, and the solid line (bias-corrected) indicates calibration when the model is applied to the bootstrap set.

**Figure 5:**
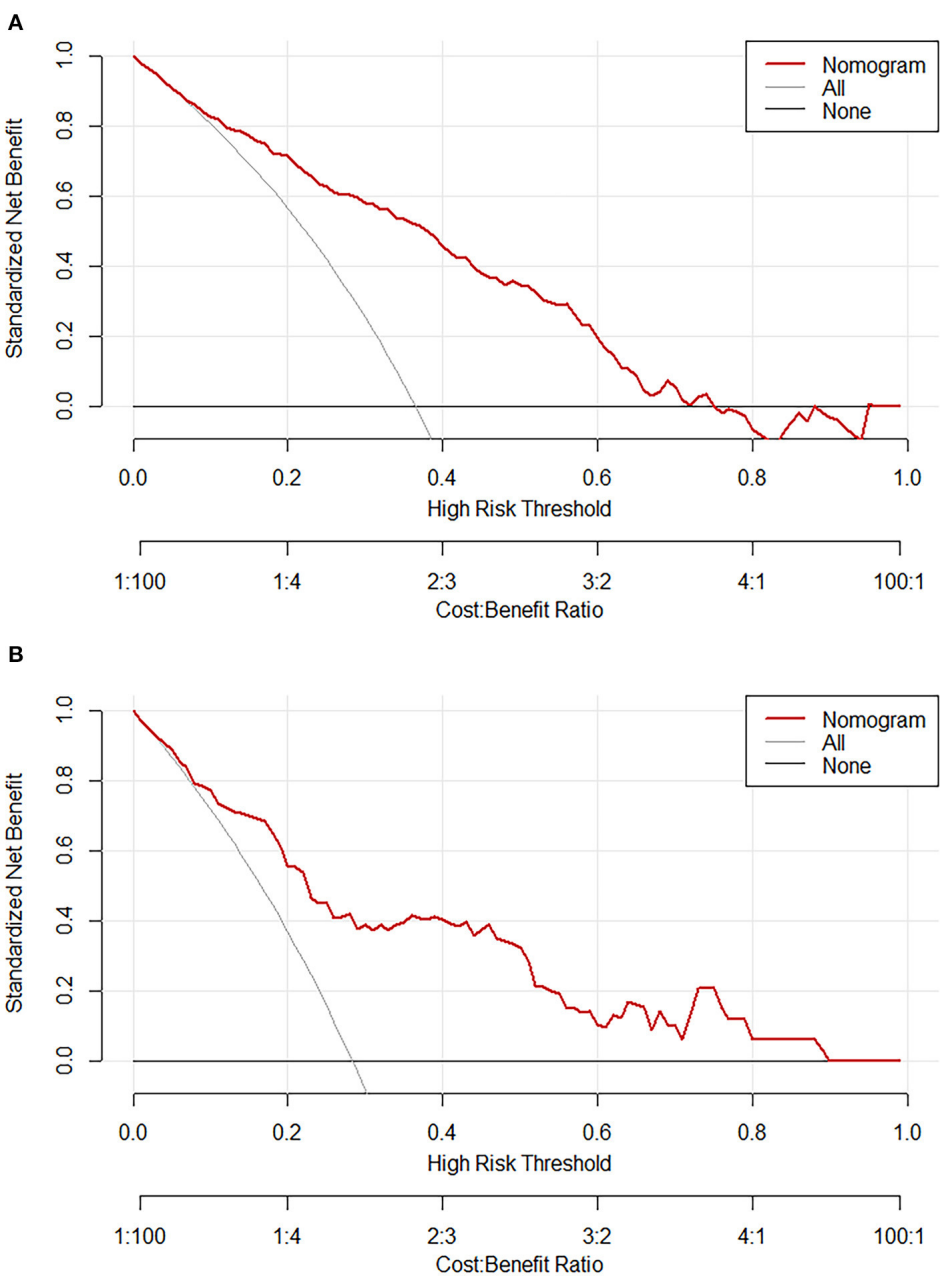
Decision curve analysis of the nomogram in the primary cohort **(A)** and validation **(B)** cohort. The red line displays the net benefit of our model. The gray line assumes that all patients develop lower extremity deep vein thrombosis (DVT). The black line assumes that no patients develop lower extremity DVT.

The calibration plot with 1,000 bootstraps and the Brier score were further used to examine the calibration performance of the nomogram. The calibration plots for the probability of lower extremity DVT presented an optimal agreement between the prediction and observation, both in the primary cohort (Figure 4A) and the validation cohort (Figure 4B). The mean absolute error values of the two calibration plots were 0.014 and 0.039, separately. We used the Brier score to further quantify the performance of the calibration; the Brier scores were 0.167 in the primary cohort and 0.183 in the validation cohort. The decision curve analysis was plotted to measure the clinical benefit of the nomogram. Figures 5A,B show the two decision curve analyses of the primary and validation cohort, which illustrated that patients could benefit from the nomogram.

## Discussion

In this prospective observational study, age, GCS score, D-dimer level, muscle strength, and infection were identified as independent predictors of lower extremity DVT in neurointensive care unit patients. Based on the five variables, the nomogram model was developed to assess the risk of lower extremity DVT in neurointensive care unit patients. Our internal validation showed that it was discriminatory and well-calibrated, and external validation showed satisfactory accuracy and generalizability.

This study found that in our neurointensive care unit patient cohort, the incidence of lower extremity DVT was 36.4%, which was higher than the incidence in patients after neurosurgery who are not admitted to the neurointensive care unit reported by other studies (1, 3). This may be because intensive care unit patients have a higher risk of DVT overall (9–11). Among the 420 patients included in the primary cohort, the age of patients with lower extremity DVT was significantly higher than those without (*P* < 0.001). Logistic regression analysis demonstrated that age (OR = 1.050, 95% CI: 1.029–1.071) was an independent predictor for lower extremity DVT (1, 12). Previous studies confirmed that elderly patients often have poor vascular elasticity, rough vascular intima, and decreased muscle pump function; most patients in this age group also have complications such as hypertension, diabetes, and hyperlipidemia, which promote vascular endothelial damage, resulting in an increased incidence of DVT (1, 3, 12, 13).

This study revealed that the GCS scores of the patients who developed lower extremity DVT were significantly lower than those who did not develop lower extremity DVT (*P* < 0.001). Furthermore, multivariate analysis showed that a high GCS score (OR = 0.889, 95% CI: 0.825–0.959) was a protective factor for lower extremity DVT. This may be because low GCS scores are often associated with consciousness disorders, limb movement disorders, and a long-term bedridden state, which leads to slow blood circulation and increases the risk of DVT for these patients (3, 12, 14).

Previous studies have shown that paralysis can lead to the slowing of blood flow and vascular stasis in the affected limb, and paralysis carries a high risk for lower extremity DVT (3, 15). By using the measure of muscle strength, this study expands on this knowledge by showing that the risk of lower extremity DVT in patients with muscle strength ≤ grade 3 was 1.4 times higher (95% CI: 1.346–4.366) than that in the patients with muscle strength ≥ grade 4. Therefore, it is necessary to provide timely assessment and intervention measures for patients with paralysis or with muscle strength of grade 3 and below.

In our study, infection was another major predictor of lower extremity DVT for neurointensive care unit patients. Many studies have confirmed that infection could not only cause direct injury to vascular intima, but also release a series of inflammatory mediators, leading to abnormal coagulation and activating the external coagulation system, thus increasing the risk for lower extremity DVT (16, 17).

In addition, the D-dimer level has been previously confirmed as a sensitive indicator to predict the occurrence of venous thromboembolism in patients (2, 18, 19). Our results further supported that the D-dimer level has a reference value for the early judgment of lower extremity DVT (OR = 1.040, 95% CI: 1.008–1.074). This suggests that we should dynamically detect and evaluate the results of D-dimer levels to identify and monitor the occurrence of DVT at an earlier stage.

This study has certain clinical significance for neurointensive care unit medical staff and patients: early administration of anticoagulants remains a controversial issue, as some researchers have indicated that early pharmacological prophylaxis increases the risk of bleeding (20, 21), whereas others argued that early use of anticoagulants is not associated with late bleeding (22, 23); furthermore, there are no clear guidelines for the application of anticoagulants in neurointensive care unit patients with different diagnoses; however, the prognostic nomogram model may assist neurosurgeons in the early identification of high-risk patients to provide them with active individual anticoagulant treatment after cautious assessment of thrombosis vs. bleeding.

Nevertheless, our study has several limitations. First, the samples of the study were obtained from one tertiary hospital in Shanghai and so was limited as a single-center study. *Second*, data collection was performed before the COVID-19 pandemic; however, the latest study from the COVIDSurg Collaborative group demonstrated that SARS-CoV-2 infection was also a significant predictor of DVT in critically ill patients (24). Therefore, it will be more meaningful to explore the optimal prophylaxis and treatment protocols for DVT in combination with the above risk factors in the setting of the COVID-19 pandemic. Additionally, several risk factors reported by previous studies were not involved in this study, such as malignant tumor (1, 2), application of dehydration drugs (2), and femoral CVC (in this study, we did not distinguish the superior vena cava catheter from the femoral CVC, which may have led to the femoral CVC being identified as a non- significant variable) (13, 25). Lastly, although we found that age and D-dimer level were independent risk factors for DVT, we did not further explore the age and D-dimer thresholds that could indicate lower extremity DVT in neurointensive care unit patients. In the future, we will look forward to more multicenter and larger prospective cohort studies to further improve and validate the model.

## Conclusions

Age, GCS score, muscle strength ≤ grade 3, infection, and D-dimer level are the major predictors of lower extremity DVT in neurointensive care unit patients. The discrimination, accuracy, and clinical effectiveness of the prognostic nomogram developed in this study led to satisfactory performance for predicting lower extremity DVT. It can help medical staff identify patients at high risk of lower extremity DVT in the neurointensive care unit, so targeted interventions can be administered to reduce the incidence of lower extremity DVT and its adverse complications.

## Data Availability Statement

The raw data supporting the conclusions of this article will be made available by the authors, without undue reservation.

## Ethics Statement

The studies involving human participants were reviewed and approved by Ethics Committee of Shanghai Tenth People's Hospital: NO.SHSY-IEC-4.0/19-72/02. The patients/participants provided their written informed consent to participate in this study.

## Author Contributions

LZ and XZ: conception, design, and revision of the article. RL, JZ, YW, and LW: data collection. JJ, RL, and YS: analysis and drafting of the article. XZ, LZ, JJ, JZ, RL, YS, YW, and LW: contributed to the article and approved the final version. All authors contributed to the article and approved the submitted version.

## Funding

This study was supported by the projects of the Young Scientists Fund of the National Natural Science Foundation of China (No. 72004162) and the National Natural Science Foundation of China (No. 72074168).

## Conflict of Interest

The authors declare that the research was conducted in the absence of any commercial or financial relationships that could be construed as a potential conflict of interest.

## Publisher's Note

All claims expressed in this article are solely those of the authors and do not necessarily represent those of their affiliated organizations, or those of the publisher, the editors and the reviewers. Any product that may be evaluated in this article, or claim that may be made by its manufacturer, is not guaranteed or endorsed by the publisher.
