# Supplementary material for: Prognostic Nomogram for Predicting Lower Extremity Deep Venous Thrombosis in Neurointensive Care Unit Patients: A Prospective Observational Study
Source: Front Neurol. 2022 Jan 28;12:761029. doi: 10.3389/fneur.2021.761029 (PMC8831723; doi:10.3389/fneur.2021.761029)
Supplement: Supplementary file 1 [file Data_Sheet_1.DOCX]

#### **Questionnaire of patients** **in neurointensive care unit**

| Patient ID: | Name: |
| --- | --- |
| **(1) Demographic features** | |
| Gender: | Age: |
| **(2) Past medical history (Yes/No)** | |
| Hypertension: | Diabetes mellitus: |
| **(3) Clinical features (fill these blanks at entering the neurointensive care unit)** | |
| Diagnose: Neurovascular disease □ / Central nervous system tumor □  Traumatic brain injury □ / Others □ | |
| GCS score: | |
| Caprini score: | Muscle strength grade (0-V): |
| APACHE-II score (total): | |
| APACHE-IIA: | APACHE-IIB: |
| APACHE-IIC: | APACHE-IID: |
| **(4) Clinical features (fill the blank at the third day of** **surgery or conservative treatment)** | |
| D-dimer levels(μg/mL): | |
| **(5) Clinical features (fill these blanks if the patients lower extremity DVT positive or out of department)** | |
| Lower extremity deep venous thrombosis | Yes □ / No □ |
| Calf muscle vein thrombosis | Yes □ / No □ |
| Pulmonary embolism | Yes □ / No □ |
| Sedative drugs | Yes □ / No □ |
| Hemostatic drugs | Yes □ / No □ |
| Vasopressors | Yes □ / No □ |
| Central venous catheter | Yes □ / No □ |
| Surgery | Yes □ / No □ |
| Infection: Intracranial infection □ / Urinary tract infection □  Catheter-related bloodstream infection□ / Pneumonia □ | |
| Mechanical ventilation (hours): | |
| Neurointensive care unit stay (days): | |

Nurse name: Date:
